# Supplementary material for: Piezo1/2 mediate mechanotransduction essential for bone formation through concerted activation of NFAT-YAP1-ß-catenin
Source: eLife. 2020 Mar 18;9:e52779. doi: 10.7554/eLife.52779 (PMC7112954; doi:10.7554/eLife.52779)
Supplement: Supplementary file 1. [file elife-52779-supp1.docx]

| Supplemental Table 1. μCT quantification of *Sp7*-*Cre* line | | | | | | | |
| --- | --- | --- | --- | --- | --- | --- | --- |
|  | Wild type (n=4) | Piezo1 CKO (n=3) | | Piezo2 CKO (n=6) | | Piezo1/2 DKO (n=3) | |
|  | Means ± SD | Means ± SD | *P* value^A^ | Means ± SD | *P* value^A^ | Means ± SD | *P* value^A^ |
| Ct. BMD (mgHA/cm^3^) | 1004 ± 4.103 | 987.1 ± 5.238 | 0.0505 | 986.9 ± 6.556 | 0.2332 | 966.9 ± 7.833 | 0.0133 |
| Ct. Th (mm) | 0.097 ± 0.003 | 0.084 ± 0.003 | 0.028 | 0.096 ± 0.001 | 0.9868 | 0.084 ± 0.004 | 0.0326 |
| Tb. Sp (mm) | 0.429 ± 0.050 | 2.11 ± 0.294 | <0.0001 | 0.572 ± 0.059 | 0.7834 | 2.956 ± 0.046 | <0.0001 |
| Tb. N (1/mm) | 2.243 ± 0.266 | 0.489 ± 0.078 | 0.0006 | 1.748 ± 0.195 | 0.2911 | 0.304 ± 0.036 | 0.0002 |
| Tb. BV/TV (%) | 7.588 ± 0.926 | 1.373 ± 0.220 | 0.0011 | 5.910 ± 0.785 | 0.3802 | 0.777 ± 0.058 | 0.0005 |
| Ct.BMD: Bone mineral density of cortical bone; Ct.Th: Thickness of cortical bone; Tb.Sp: Separation of trabecular bone; Tb.N: Number of trabecular bone; Tb.BV/TV: Ratio of bone volumn and total volumn of trabecular bone. Two-tailed unpaired Student’s *t*-test and one-way ANOVA followed by Tukey’s multiple comparisons tests when ANOVA was significant. A: Compare with wild type. | | | | | | | |
| Source data 14--Table S1. | | | | | | | |
