# Supplementary material for: Piezo1/2 mediate mechanotransduction essential for bone formation through concerted activation of NFAT-YAP1-ß-catenin
Source: eLife. 2020 Mar 18;9:e52779. doi: 10.7554/eLife.52779 (PMC7112954; doi:10.7554/eLife.52779)
Supplement: Supplementary file 2. [file elife-52779-supp2.docx]

**Supplemental Table 2**

| **Gene name (mouse)** | **direction** | **Sequence** |
| --- | --- | --- |
| ***Mus musculus*** | | |
| *Gapdh* | Forward primer | 5′-AGGTCGGTGTGAACGGATTTG-3′ |
|  | Reverse primer | 5′- TGTAGACCATGTAGTTGAGGTCA-3′ |
| *Alpl* | Forward primer | 5′-CTTGACTGTGGTTACTGCTGAT-3′ |
|  | Reverse primer | 5′-GGAATGTAGTTCTGCTCATGGA-3′ |
| *Sp7* | Forward primer | 5′-CCCACTGGCTCCTCGGTTCTCTCC-3′ |
|  | Reverse primer | 5′-GCTBGAAAGGTCAGCGTATGGCTTC-3′ |
| *Col1a1* | Forward primer | 5′-CACCCTCAAGAGCCTGAGTC-3′ |
|  | Reverse primer | 5′-GTTCGGGCTGATGTACCAGT-3′ |
| *Spp1* | Forward primer | 5′-GGCATTGCCTCCTCCCTC-3′ |
|  | Reverse primer | 5′-GCAGGCTGTAAAGCTTCTCC-3′ |
| *Runx2* | Forward primer | 5’-AACCCACGGCCCTCCCTGAACTCT-3’ |
|  | Reverse primer | 5’-ACTGGCGGGGTGTAGGTAAAGGTG-3’ |
| *Ibsp* | Forward primer | 5′-TACCGGCCACGCTACTTTCTTTAT-3′ |
|  | Reverse primer | 5′-GACCGCCAGCTCGTTTTCATCC-3′ |
| *Bglap* | Forward primer | 5′-ACCCTGGCTGCGCTCTGTCTCT-3′ |
|  | Reverse primer | 5′-GATGCGTTTGTAGGCGGTCTTCA-3′ |
| *Dmp1* | Forward primer | 5′-ATTTGGCTGGGTCACCACCA-3′ |
|  | Reverse primer | 5′-CAGATTCACTGCTGTCCGTGTG-3′ |
| *Tnfsf11* | Forward primer | 5’-CACAGCGCTTCTCAGGAGCTC-3’ |
|  | Reverse primer | 5’-GAGATCTTGGCCCAGCCTCGA-3’ |
| *Acp5* | Forward primer | 5’-AGTGCACGAGCCAGCGACAA-3’ |
|  | Reverse primer | 5’-CCAGCGCCTGGAGATCTTAGA-3’ |
| *Tnfrsf11b* | Forward primer | 5’-AGTCCGTGAAGCAGGAGTGCA-3’ |
|  | Reverse primer | 5’-AAGTCTCACCTGAGAAGAACC-3’ |
| *Yap1* | Forward primer | 5’-GACTCCGAATGCAGTGTCTTC-3’ |
|  | Reverse primer | 5’-TGTTGAGGAAGTCGTCTGGG-3’ |
| *Lef1* | Forward primer | 5′-CTTCGCCGAGATCAGTCATCC -3′ |
|  | Reverse primer | 5′-ACGGGTCGCTGTTCATATTGG-3′ |
| *Tcf7* | Forward primer | 5′-TCGAGAAGAGCAGGCCAAGT-3′ |
|  | Reverse primer | 5′-AGAGCACTGTCATCGGAAGGAA-3′ |
| *Axin2* | Forward primer | 5′- CCATTGGAGTCTGCCTGTG-3′ |
|  | Reverse primer | 5′- GGACACTTGCCAGTTTCTTTG-3′ |
| *Ccn2* | Forward primer | 5′- CTGCCTACCGACTGGAGAC -3′ |
|  | Reverse primer | 5′-CATTGGTAACTCGGGTGGAG.-3′ |
| *Ccn1* | Forward primer | 5′- GCTCAGTCAGAAGGCAGACC -3′ |
|  | Reverse primer | 5′-GTTCTTGGGGACACAGAGGA-3′ |
| *Piezo1* | Forward primer | 5′-TCATCATCCTTAACCACATGGTG-3′ |
|  | Reverse primer | 5′-TGAAGACGATAGCTGTCATCCA-3′ |
| *Piezo2* | Forward primer | 5′-GTGGTATGCAACCCAGTACCC-3′ |
|  | Reverse primer | 5′-GGCCATTCTCTATGGGCAGG-3′ |
| ***Homo sapiens*** | | |
| *GAPDH* | Forward primer | AGCCACATCGCTCAGACAC |
|  | Reverse primer | GCCCAATACGACCAAATCC |
| *PIEZO1* | Forward primer | CCACCAACCTCATCAGCGACTT |
|  | Reverse primer | ACCAGCACCAGCCAGAACAG |
| *PIEZO2* | Forward primer | GCCCAACAAAGCCAGTTGAA |
|  | Reverse primer | GGGCTGATGGTCCACAAAGA |
