# Supplementary material for: Piezo1/2 mediate mechanotransduction essential for bone formation through concerted activation of NFAT-YAP1-ß-catenin
Source: eLife. 2020 Mar 18;9:e52779. doi: 10.7554/eLife.52779 (PMC7112954; doi:10.7554/eLife.52779)
Supplement: Transparent reporting form [file elife-52779-transrepform.docx]

***eLife’s* transparent reporting form**

We encourage authors to provide detailed information *within their submission* to facilitate the interpretation and replication of experiments. Authors can upload supporting documentation to indicate the use of appropriate reporting guidelines for health-related research (see [EQUATOR Network](http://www.equator-network.org/%20)), life science research (see the [BioSharing Information Resource](https://biosharing.org/)), or the [ARRIVE guidelines](http://www.plosbiology.org/article/info:doi/10.1371/journal.pbio.1000412) for reporting work involving animal research. Where applicable, authors should refer to any relevant reporting standards documents in this form.

If you have any questions, please consult our Journal Policies and/or contact us: [editorial@elifesciences.org](mailto:editorial@elifesciences.org).

**Sample-size estimation**

- You should state whether an appropriate sample size was computed when the study was being designed
- You should state the statistical method of sample size computation and any required assumptions
- If no explicit power analysis was used, you should describe how you decided what sample (replicate) size (number) to use

Please outline where this information can be found within the submission (e.g., sections or figure legends), or explain why this information doesn’t apply to your submission:

We performed Power Analysis to estimate the number of mice needed for our studies. Based on our previous experiences, the standard deviation (SD) within bone changes was approximately 15%. We used the Pass software to perform the sample size calculations and power analyses (PASS 2008 Power Analysis and Sample Size for Windows, 2008, NCSS. Kaysville, Utah). Our goal is to detect a 25% difference between the control group and experimental group. With a type I error of 0.05 (Significance level), type II error of 0.2 (or equivalently power of 80%) and a two-sided test, a sample size of 8/group will enable us to detect a 25% difference between the control and experimental groups. Bigger difference between groups and less variation within a group will reduce the sample size. For genetic knockout models, at least n=3 mice per group from 2 independent litters were analyzed. The exact sample size is listed in the figure legends.

**Replicates**

- You should report how often each experiment was performed
- You should include a definition of biological versus technical replication
- The data obtained should be provided and sufficient information should be provided to indicate the number of independent biological and/or technical replicates
- If you encountered any outliers, you should describe how these were handled
- Criteria for exclusion/inclusion of data should be clearly stated
- High-throughput sequence data should be uploaded before submission, with a private link for reviewers provided (these are available from both GEO and ArrayExpress)

Please outline where this information can be found within the submission (e.g., sections or figure legends), or explain why this information doesn’t apply to your submission:

A technical replicate is repetition of the same experiment with the same sample multiple times. It's used to test the variability in the testing protocol itself. A biological replicate is to perform the same test on multiple samples of the same material / type of cells / tissue. The samples are different, but are expected to be very similar (if not identical) with regard to the test. Biological replicates are used to test the variability between samples that were selected on the basis of being otherwise identical.

All replications shown are biological replications. All in vitro cell culture experiments were performed three times independently. Ex vivo organ cultures were performed three times with samples collected from 3 independent litters. In some studies, each experiment was replicated independently at least 3 times by multiple investigators (technical replication) to make sure that the experimental procedures are consistent and reliable. No data was excluded. RNAseq source data for Figure. S3 has been deposited in GEO under the accession number GSE139121. The secure token of GSE 139121 is sfgnywqghpejpax.

**Statistical reporting**

- Statistical analysis methods should be described and justified
- Raw data should be presented in figures whenever informative to do so (typically when N per group is less than 10)
- For each experiment, you should identify the statistical tests used, exact values of N, definitions of center, methods of multiple test correction, and dispersion and precision measures (e.g., mean, median, SD, SEM, confidence intervals; and, for the major substantive results, a measure of effect size (e.g., Pearson's r, Cohen's d)
- Report exact p-values wherever possible alongside the summary statistics and 95% confidence intervals. These should be reported for all key questions and not only when the p-value is less than 0.05.

Please outline where this information can be found within the submission (e.g., sections or figure legends), or explain why this information doesn’t apply to your submission:

The statistical analysis methods were described in the figured legends and Methods section. The exact sample size and p-values were described in the related figure legends.

(For large datasets, or papers with a very large number of statistical tests, you may upload a single table file with tests, Ns, etc., with reference to sections in the manuscript.)

**Group allocation**

- Indicate how samples were allocated into experimental groups (in the case of clinical studies, please specify allocation to treatment method); if randomization was used, please also state if restricted randomization was applied
- Indicate if masking was used during group allocation, data collection and/or data analysis

Please outline where this information can be found within the submission (e.g., sections or figure legends), or explain why this information doesn’t apply to your submission:

For basal skeletal phenotype analysis, mice were grouped based on their genotype. For BOTOX administration, mice were grouped based on their genotype, right tibiae were injected with BOTOX as unloading model, and left tibiae served as normal loading controls. This information can be found in related figure legends and Methods section.

**Additional data files (“source data”)**

- We encourage you to upload relevant additional data files, such as numerical data that are represented as a graph in a figure, or as a summary table
- Where provided, these should be in the most useful format, and they can be uploaded as “Source data” files linked to a main figure or table
- Include model definition files including the full list of parameters used
- Include code used for data analysis (e.g., R, MatLab)
- Avoid stating that data files are “available upon request”

Please indicate the figures or tables for which source data files have been provided:

RNAseq source data for Figure. S3 has been deposited in GEO under the accession number GSE139121.
